# Advances of mesenchymal stem cells and their derived extracellular vesicles as a promising therapy for acute respiratory distress syndrome: from bench to clinic

**DOI:** 10.3389/fimmu.2023.1244930

**Published:** 2023-08-29

**Authors:** Xiaoli Zhuang, Yu Jiang, Xiaofang Yang, Lin Fu, Lan Luo, Ziyuan Dong, Ju Zhao, Feilong Hei

**Affiliations:** Department of Cardiopulmonary Bypass, Beijing Anzhen Hospital, Capital Medical University, Beijing, China

**Keywords:** ARDS, ALI, MSCs, extracellular vesicles, cell-based therapy

## Abstract

Acute respiratory distress syndrome (ARDS) is an acute inflammatory lung injury characterized by diffuse alveolar damage. The period prevalence of ARDS was 10.4% of ICU admissions in 50 countries. Although great progress has been made in supportive care, the hospital mortality rate of severe ARDS is still up to 46.1%. Moreover, up to now, there is no effective pharmacotherapy for ARDS and most clinical trials focusing on consistently effective drugs have met disappointing results. Mesenchymal stem cells (MSCs) and their derived extracellular vesicles (EVs) have spawned intense interest of a wide range of researchers and clinicians due to their robust anti-inflammatory, anti-apoptotic and tissue regeneration properties. A growing body of evidence from preclinical studies confirmed the promising therapeutic potential of MSCs and their EVs in the treatment of ARDS. Based on the inspiring experimental results, clinical trials have been designed to evaluate safety and efficacy of MSCs and their EVs in ARDS patients. Moreover, trials exploring their optimal time window and regimen of drug administration are ongoing. Therefore, this review aims to present an overview of the characteristics of mesenchymal stem cells and their derived EVs, therapeutic mechanisms for ARDS and research progress that has been made over the past 5 years.

## 1 Introduction

Acute respiratory distress syndrome (ARDS) is an acute respiratory failure caused by various pathogenic factors from pulmonary and non-pulmonary sources, among which pneumonia, aspiration of gastric contents and sepsis are the most common causes (1). The histopathological feature of ARDS is diffuse alveolar damage characterized by increased alveolocapillary permeability and hyaline-membrane formation due to an uncontrolled inflammatory response mediated by innate immune cells and their derived proinflammatory cytokines, also known as a “cytokine storm” in the lungs (1, 2). ARDS is one of the most severe complications of COVID-19, an emerging infectious disease triggered by the novel severe acute respiratory syndrome coronavirus 2 (SARS-CoV-2). It has been reported that approximately 42% patients with COVID-19 pneumonia develop ARDS (3). However, due to limited medical resources for ARDS diagnosis in some low-income countries and the fact that many patients with diffuse lung injury supported with high-flow nasal cannula do not meet the ARDS Berlin definition, the incidence of ARDS appears to be underestimated (1, 2). Although great progress has been made in supportive care and ventilator management, the mortality rate among ICU and hospital patients with ARDS is still high, reaching up to 35.3% and 40.0%, respectively (4). COVID-19 ARDS seems to have a higher mortality rate compared to ARDS from other causes (5). Other than that, survivors of ARDS often experience long-term complications, including functional impairments such as muscle wasting and weakness, as well as neurocognitive disorders, which significantly compromise their quality of life (QOL) (6–8). Thus, ARDS has been a major health problem and a difficult area of clinical treatment that need to be addressed all over the world. However, at present, there is no effective pharmacotherapy available for ARDS worldwide, and the treatment is primarily symptomatic and supportive with lung-protective ventilation, fluid-conservative management strategy and prone positioning despite the intense research (9). Although various pharmacotherapies, such as surfactant replacement, β-agonists, inhaled nitric oxide, statins and activated protein C, etc. have shown promise in animal models and preclinical researches, translating these findings into effective therapies for human ARDS has proven challenging and most clinical trials focusing on consistently effective drugs have met disappointing results (7). For patients with moderate to severe ARDS, when conventional treatment approaches failed to improve their clinical conditions, extracorporeal membrane oxygenation (ECMO) is considered as a rescue therapy and can serve as a bridge to recovery (10).

In recent years, mesenchymal stem cells (MSCs) as a cell-based therapy for various inflammatory diseases due to their potent anti-inflammatory, anti-apoptotic and tissue regeneration properties had spawned intense interest of a wide range of researchers and clinicians. A growing body of evidence highlights the potential benefits of stem cell-based therapy for ARDS (11). Animal models have demonstrated that MSCs possess lung protective capacities through suppressing inflammatory responses and attenuating pulmonary edema (12–14). Furthermore, preliminary clinical trials also have displayed encouraging therapeutic outcomes of MSCs for ARDS. However, stem cell-based therapy exists some worrying issues, for example, low stability, the risk of pulmonary embolism, and tumor formation (15, 16). Recent studies have revealed that the anti-inflammatory effect of MSCs is mainly achieved *via* paracrine pathway (17). The MSCs secretome such as extracellular vesicles (EVs) has been confirmed to be the main mediators of their therapeutic attributes (18). Compared with their original stem cells, EVs possess several attractive features like targeted delivery, lower immunogenicity and tumorigenicity, easy storage, high stability, and ability to cross blood barriers. Therefore, a large number of preclinical studies on the treatment of ARDS with MSCs-derived EVs have emerged recent years. Animal experimental results were exciting. And several relevant clinical studies are also ongoing.

This review aims to make an overview about the characteristics of MSCs and their derived EVs, therapeutic mechanisms for ARDS and research progress that has been made over the past 5 years. We will not focus on the pathogenesis of ARDS in this review, as it has been extensively covered in other published reviews (1, 2, 19). Finally, we will briefly summarize the possible future directions of MSCs and their derived EVs for ARDS therapy.

## 2 Overview of mesenchymal stem cells and their derived extracellular vesicles

**Figure 1:**
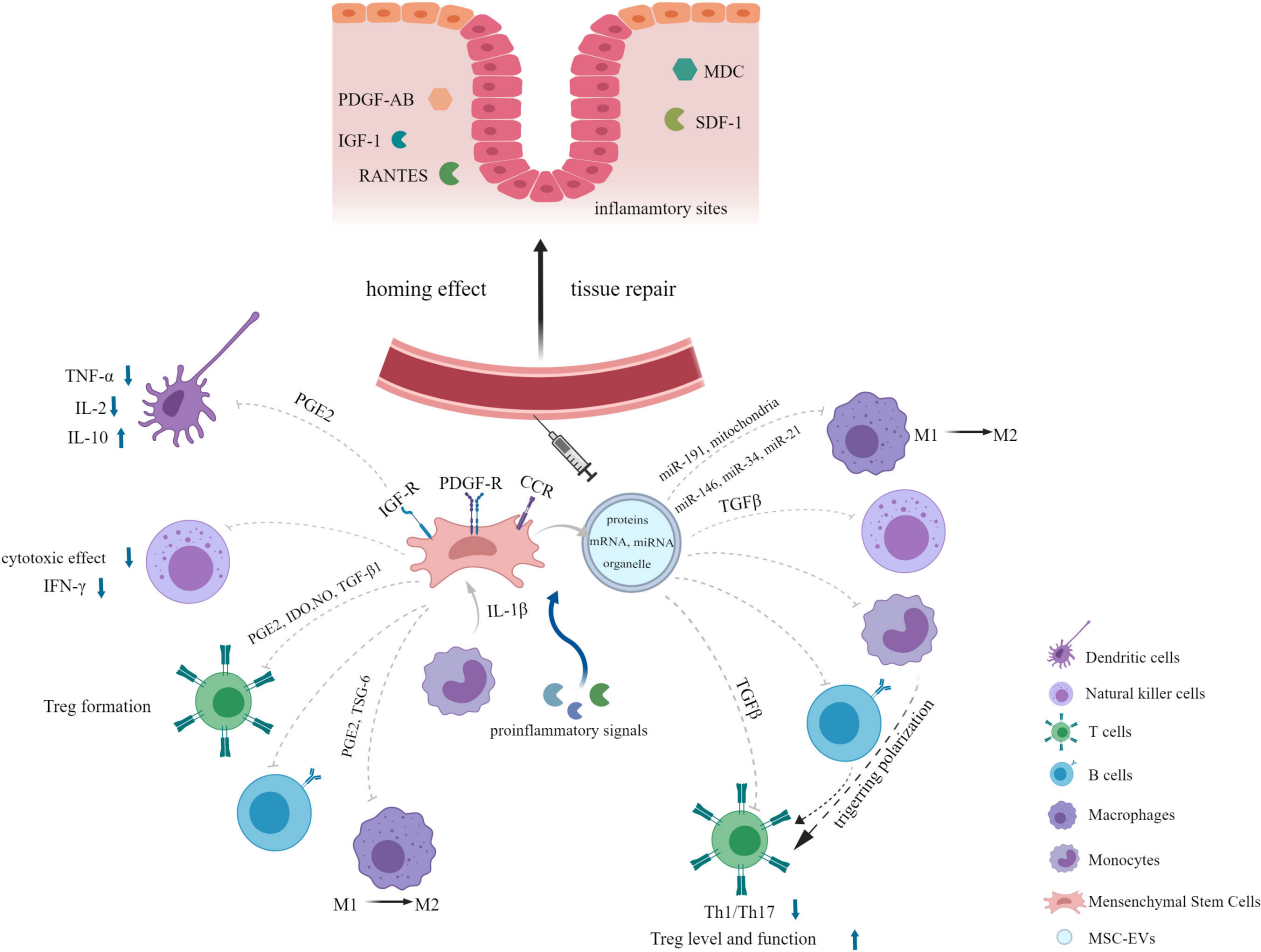
Overview of the characteristics of MSCs and their derived EVs in inflammatory diseases.

Stem cell-based therapy has become a hotspot in the field of regenerative medicine. Among various types of stem cells, MSCs are evoking the most extraordinary interest due to their advantages of simplicity of isolation, low immunogenicity and tumorigenicity, high proliferation rate and free of ethical issues (11, 20–22). Additionally, EVs secreted by MSCs have the same effects as their parental cells. In this section, we will provide an overview about the characteristics of MSCs and their derived EVs, shedding light on their potential in inflammatory diseases (**Figure 1**).

### 2.1 Mesenchymal stem cells

The concept of MSCs originated from the non-hematopoietic stem cells present in bone marrow (BM) (23). Researches on MSCs can be traced to 1960s, when Tavassoli and Crosby clearly established the proof of bone and marrow generation in *de novo* potential of BM transplanted to extramedullary site (23, 24). Subsequently, a series of seminal studies conducted by Friedenstein and his colleagues demonstrated that only a small subset of BM cells was responsible for the osteogenic potential of BM allograft (25). Along with the development of hematopoietic stem cell research, the groundbreaking work from Tavasolli, Friedenstein, and Owen revealed possible existence of a second type of stem cell in bone marrow (23). The term “mesenchymal stem cells” was first coined by Caplan in 1991 (26). However, it was not until 1999 that the term gained widespread popularity (23, 27).

MSCs are considered as a heterogeneous subpopulation of adult multipotent cells with self-renewing and differentiation potential (28). MSCs show a fibroblast-like morphology under the microscope. According to the minimal criteria to define human MSCs proposed by the International Society for Cellular Therapy in 2006, MSCs must meet the following three criteria: 1) plastic adherence in standard culture conditions; 2) expression of specific surface antigens such as CD105, CD73, and CD90, but lacking expression of CD45, CD34, CD14 or CD11b, CD79α or CD19, and HLA-DR; 3) trilineage differentiation into osteocytes, chondrocytes and adipocytes *in vitro (* 29). In addition to bone marrow, MSCs can also be isolated from the other multiple tissues, including adipose tissue (30), placenta (31), umbilical cord (32), Wharton’s jelly (33), dental pulp (34), periodontal ligament (35), synovial membrane (36), periosteum (37), skin (38), skeletal muscle (39), tonsil (40), amniotic fluid (41), uterine cervix (42), peripheral blood (43), among which bone marrow-derived mesenchymal stem cells (BM-MSCs) are studied most extensively (44).

MSCs have been shown to be a promising therapy for various diseases such as autoimmune disease (45), graft-versus-host disease (GvHD) (46), inflammatory disease (47), neurodegenerative disorder (48), cardiovascular disease (49), wound healing repair (50) and diabetes mellitus (51) due to their tissue repair and immunomodulatory properties (21, 52). One of the important properties that make MSCs play therapeutic role in diseases mentioned above is their capacity of migration to injured or inflammatory sites when injected systematically, which is called the homing effect (44, 53). Migration of MSCs occurs in response to a large amount of chemotactic factors, including both growth factors (GFs) and chemokines generated in the damaged tissues, such as platelet-derived growth factor-AB (PDGF-AB), insulin-like growth factor 1 (IGF-1), RANTES (regulated upon activation, normal T-cell expressed and secreted), macrophage-derived chemokine (MDC), and stromal-derived factor-1 (SDF-1) (54, 55). Meanwhile, a myriad of relevant receptors expressed on MSCs membranes have been detected, including tyrosine kinase receptors PDGF-receptor (R) α, PDGF-Rβ, and IGF-R and chemokine receptors CCR3, CCR4, CCR5, CXCR4 (55). Later, the surface proteins podocalyxin-like protein (PODXL) and α6-integrin (CD49f) highly expressed in the early cultures of MSCs have been found to enhance their migration to injured tissues after systemic administration (56). Adhesion molecules and integrins such as VCAM-1 and VLA-4 contribute to the migration of MSCs to the inflammatory sites as well (57). In targeted location, MSCs can mediate productive repair by secreting bioactive factors with the capacity of anti-apoptotic, immunomodulatory and angiogenic effects and interacting with injured host cells (12, 52, 58–60). Another reason for the widespread use of MSCs is its robust immunomodulatory property. MSCs exert immunomodulatory effects by suppressing innate and adaptive immune responses, which involve a variety of immune cells. MSCs have been demonstrated to not only interfere with all major stages of the dendritic cells (DCs) life cycle: differentiation, maturation, and activation, resulting in the formation of immature DCs with an inhibitory phenotype but also alter the cytokine secretion profile of DCs, as evidenced by decreased secretion of tumor necrosis factor α (TNF-α) and interleukin-2 (IL-2) and increased IL-10 secretion (17, 61, 62). In addition, MSCs inhibit the cytotoxic effects of natural killer (NK) cells and interferon γ (IFN-γ) secretion. They also modulate immune responses bysuppressing T lymphocyte activation and proliferation. MSCs can induce T-cell anergy and promote the formation of regulatory T cells (Tregs). They enable CD4+ T cells to generate a functional Treg population but suppress the activation, proliferation and differentiation of proinflammatory T cells during the CD4+ T cells induced to differentiate toward Th1 or Th17 cells (63). Furthermore, differentiation, antibody production, and chemotactic behavior of B cells are affected by MSCs as well (17). The exact mechanism underlying this immunosuppressive effect of MSCs is not well understood, but it is believed to involve soluble factors secreted by MSCs, such as prostaglandin E2 (PGE2) (61), tryptophan catabolizing enzyme indoleamine 2,3-dioxygenase (IDO) (64), nitric oxide (NO) (65) and transforming growth factor-β1 (TGF-β1) (66). Notably, there are complex interactions between antigen-presenting cells (APCs), adaptive immune cells, and MSCs. APCs, especially blood CD14+ monocytes, play a critical role in activating MSCs through the release of IL-1β, which then enables MSCs to inhibit T lymphocytes (66).

The mechanism of MSC-mediated immunosuppression varies among different species. For instance, a study demonstrated that MSCs from human and non-human primates mediated immunosuppression by IDO whereas MSCs from mouse by NO (67). Moreover, the immunosuppressive ability of MSCs may not be inherent, but rather is elicited in the presence of proinflammatory cytokines and is chemokine-dependent (46, 67). When exposed to certain combinations of cytokines, MSCs produce high levels of inducible nitric oxide synthases (iNOS) and chemokines which drive immune cells migration into proximity to MSCs, where immune cell function can be suppressed by the high levels of NO (46). Proinflammatory signals activated MSCs to secret PGE2 and TNFα stimulated gene/protein 6 (TSG-6), both of which are negative feedback loops of inflammation, leading to a shift in macrophages from the M1 phenotype (proinflammatory) to the M2 phenotype (anti-inflammatory) (68). MSCs can also be activated by microenvironment generated by injured tissues to express factors that appear to specifically meet the needs of the tissues (69). Special storage conditions, low cell engraftment and poor survival in the lung, and cell senescence during *in vitro* expansion are the main obstacles limiting MSCs clinical translation (15, 20, 70, 71). However, several strategies have been developed to optimize MSCs therapy in ARDS, such as genetic modification and pretreatment with a series of preconditioning strategies (20), which will be discussed in detail in the section of functional optimization of MSCs and their derived EVs.

### 2.2 MSC-derived extracellular vesicles

It is now widely accepted that the therapeutic potential of MSCs is largely attributed to their secretome, especially their derived EVs. EVs are nanometer sized particles secreted by almost all cell types. Previously, EVs were generally classified into exosomes, microvesicles, and apoptotic bodies according to their size and intracellular origin (18). The Minimal Information for Studies of Extracellular Vesicles 2018 (MISEV2018) guidelines updated the nomenclature of EVs since there has not yet consensus on specific markers of EVs subtypes. Therefore, based on the physical characteristics such as size, EVs can be categorized into small EVs (sEVs) with a diameter <100nm or 200nm, and medium/large EVs (m/lEVs) with a diameter>200nm (72). MSC-derived EVs express some specific surface molecules reflecting the cells of origin, such as CD29, CD73, CD44, and CD105 (18). EVs have been considered to participate in the intercellular communication *via* delivering their contents into the recipient cells, including proteins, RNA, DNA, amino acids, lipids, metabolites, and even organelles, changing the function and/or phenotype of the recipient cells under the physiological and pathological processes (73, 74). For example, insulin-like growth factor-1 receptor (IGF-1R) mRNA contained within MSC-derived EVs could be transferred to tubular cells, where it was translated in the corresponding protein, resulting in increased cell proliferation (75). EVs originated from MSCs not only exhibit similar or even superior behavior and function as MSCs but also overcome the disadvantages of live cell injection (76). As a promising cell-free therapeutic approach, MSC-derived EVs have many compelling advantages like high stability and biocompatibility, easy storage, no risk of iatrogenic tumor formation, low immunogenicity and easy to cross the biological barrier compared to their parent cells (77, 78). Over 150 miRNAs and 850 proteins have been identified from cargos of MSCs-derived EVs, which contribute to their therapeutic efficacy in a wide range of pathological conditions, including cardiovascular disease, neurological disease, acute kidney injury, liver injury and inflammatory lung diseases (74, 78–83). miRNAs are small non-coding RNAs enriched in EVs that can be internalized into the target cells and subsequently interact with specific binding sites to alter behavior of cells in physiology or pathology conditions. In recent years, with the deepening of researches on contents of MSC-derived EVs, multiple cytokines, growth factors, and prostaglandins found in EVs have been revealed to mediate immuno-modulatory properties (84).

MSC-derived EVs inherit many of the properties of their parental MSCs in the treatment of inflammatory diseases, such as immunomodulation, homing effect and tissue repair functions. After intravenous injection, MSC-derived EVs can also migrate to the injury and inflammatory sites, which may be attributed to the increased vascular permeability at the site of inflammation (76, 83, 85). As with MSCs, MSC-derived EVs display a similar immunomodulatory ability through interacting with various effector cells of the immune system (84). Co-culture of MSC-derived EVs with peripheral blood mononuclear cells (PBMCs) *in vitro* reduces PBMC proliferation (83, 86). MSC-derived EVs also modulate CD4+ T cell subsets, as evidenced by suppressing T cell induction to Th1/Th17 subtypes, while enhancing Tregs level and function significantly (83, 87–89). The proliferation, activation, and apoptosis of B lymphocytes are also inhibited (90). MSC-derived EVs can induce macrophage polarization towards the anti-inflammatory M2 phenotype (91). Moreover, it was reported that the proliferation, activation, and cytotoxicity of NK cells were inhibited by human fetal liver (FL) MSC-derived EVs (92). The underlying mechanism by which MSC-derived EVs regulate the function of CD4+ T cells and NK cells may involve TGFβ signaling (92, 93). Nevertheless, MSC-derived EVs promote Tregs immunosuppression capacity not by contacting with CD4+ T cells directly, but by triggering the polarization of T cells through interacting with APCs such as monocytes and B cells in PBMCs, which may be the mediator between MSC-derived EVs and Tregs (88). Specific miRNAs contained in MSC-derived EVs, such as miR-191, have been identified to inhibit immune response through interacting with specific proteins, such as death-associated protein kinase 1 (DAPK1) after being transferred to macrophages (94). Phinney et al. demonstrated that MSCs modulated macrophage function by transfer of partially depolarized mitochondria and regulatory microRNAs employing two different types of EVs, which enhanced macrophage bioenergetics and suppressed Toll-like receptor (TLR) signaling, respectively (95). Proinflammatory stimuli can enhance the immunosuppressive functions of MSC-derived EVs, in which multiple anti-inflammatory miRNAs, such as miR-146, miR-34, miR-21 were strongly upregulated under the stimulation of inflammatory cytokines and transferred to macrophages, resulting in M2 polarization (83, 96, 97). In addition to miRNAs, deep RNA sequencing and proteomic analysis of MSC-derived EVs revealed that several mRNAs and proteins with anti-inflammatory properties were highly enriched in cytokine-stimulated EVs compared to non-stimulated EVs (83). Anti-inflammatory mRNAs and proteins of particular importance include IDO mRNA, macrophage inhibitory cytokine 1 (MIC-1), Galectin-1 (Gal-1), and latent-transforming growth factor β-binding protein (LTBP) (83). This may explain its potent anti-inflammatory effects and therapeutic potential in various inflammatory diseases. Furthermore, some chemokines and chemokine receptors such as CCL2, VEGFC, and CCL20 carried in MSC-derived EVs can recruit immune cells to the vicinity of EVs and subsequently induce immunosuppressive effects (98). At the same time, the complex composition of EVs allows them to target different pathogenic pathways and act synergistically to enhance their therapeutic function (83).

### 2.3 Differences between MSCs and their derived EVs

It has been reported that MSCs were initially trapped by the lung and gradually accumulated in the liver and other organs after intravenous infusion, which might be due to the cellular diameter and attachment potential, while intravenously administered EVs are predominantly distributed in the spleen followed by the liver, and then the lungs and kidneys (99, 100). Regarding to the homing capability, MSCs are considered to migrate towards damaged or inflammatory sites in response to chemokines produced at the site of injury and chemokine receptors expressed on their own surface (101), while MSC-derived EVs appears to exhibit an inherent tendency towards injured tissues (102). MSC-derived EVs have many advantages over MSCs in treating various inflammatory diseases. EVs are nanoscale particles with smaller diameter, which means virtually no risk of microvascular obstruction, especially pulmonary capillaries. This feature also allows them to reach target locations faster to execute their tissue repair functions (101, 103). Besides, MSCs require special storage conditions (liquid nitrogen), while EVs are more stable and can be stored at -80°C for a long time. Amplifying stem cells *in vitro* with inappropriate processes and culture conditions may increase their immunogenicity (104). In contrast, EVs are poorly immunogenic, which enables them to prevent therapeutic cargoes from rapid degradation in the body (99). And MSC-derived EVs have increased circulating half-life (105). Moreover, MSC-derived EVs have the ability to cross the blood-tissue barrier (101). Despite these advantages, there are still many technical challenges to overcome in the process of clinical translation of MSC-derived EVs, which will be elaborated in the sixth part.

## 3 Role of mesenchymal stem cells and their derived EVs in pathophysiology of ARDS

**Figure 2:**
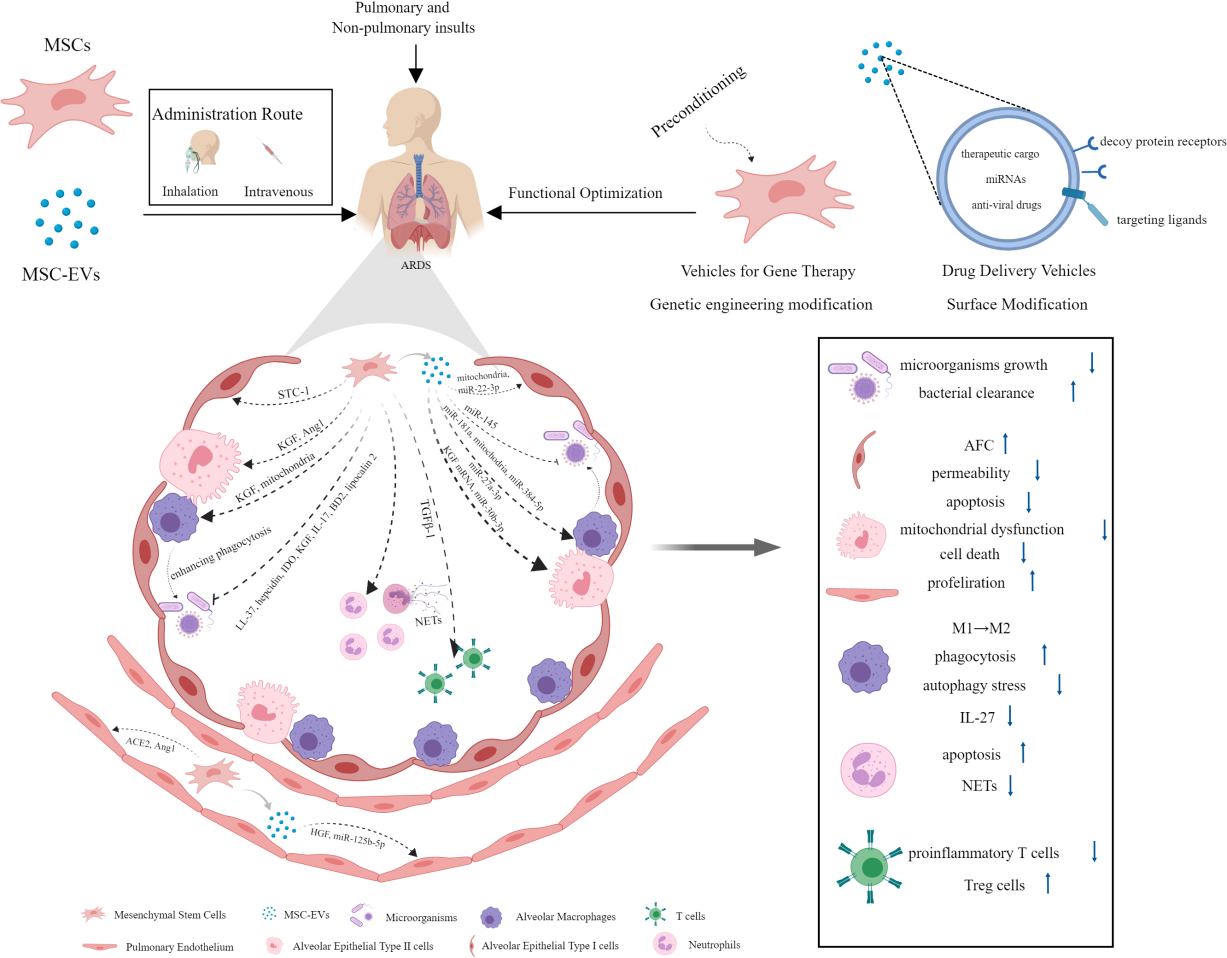
The mechanisms underlying the protective effect of MSCs and their derived EVs against ARDS.

Increasing evidence have revealed good therapeutic prospects of MSCs and their derived EVs for ALI/ARDS. They play a protective role by intervening in multiple pathogenic pathways. This part aims to make an overview of the underlying mechanisms (**Figure 2**).

### 3.1 Antimicrobial

Current data have suggested that MSCs and their EVs exert strong antimicrobial effects through indirect and direct mechanisms (106, 107). MSCs could inhibit a range of clinically relevant pathogenic microorganisms growth directly *in vivo* and *in vitro*, which was mediated in part by the secretion of antimicrobial factors, such as human cathelicidin antimicrobial peptide hCAP-18/LL-37 and hepcidin (108, 109), IDO (110), keratinocyte growth factor (KGF) (111), interleukin-17 (IL-17) (112), and β-defensin 2 (BD2) (113). In a mouse model of Gram-negative pneumonia, murine MSCs have been found to enhance bacterial clearance from the lung due to the upregulation of antibacterial protein lipocalin 2 in response to lipopolysaccharide (LPS) and inflammatory mediators (114). Besides that, the antimicrobial activity of MSCs could be achieved indirectly by enhancing alveolar macrophage (AM) phagocytosis against bacteria and the survival of blood monocytes partly through the secretion of KGF and transferring mitochondria to innate immune cells (111, 115). Macrophages of healthy individuals and those with sepsis that are exposed to MSCs have stronger ability of bacterial phagocytosis and killing (116). It has been revealed that MSC-EVs can enhance bacterial clearance in *E. coli* pneumonia mice and *ex vivo* perfused human lung models of *E. coli* pneumonia as well (117). The mechanisms underlying their antimicrobial activity could ascribe to the increased leukotriene (LT) B4 production in injured alveolus mediated by decreased multidrug resistance-associated protein 1 (MRP1) expression resulting from the transfer of miR-145 by MSC-EVs (118). The anti-microbial effect of MSC-EVs was also partly due to the enhancement of human monocytes and alveolar macrophages phagocytic capacity against bacteria (107, 117).

### 3.2 Restoration of alveolar-capillary barrier integrity

One of the pathological manifestations presented by ARDS patients is alveolar edema, which is ascribed to increased lung endothelial barrier permeability and reduced alveolar fluid clearance (AFC) (119). AFC is predominately dependent on the function of alveolar epithelial fluid transport. In experiments with perfused human lungs, treatment with human MSCs or MSC conditioned medium (MSC-CM) normalized AFC and restored lung endothelial permeability induced by *E. coli* endotoxin, thus improving pulmonary edema *via* secretion of paracrine soluble factor KGF in large part, which might traffick epithelial sodium channels subunit (αENaC) to the apical membrane of alveolar epithelial type II (AT II) cells (120). The researchers also found that human MSC-EVs were as effective as their parent stem cells in reducing lung protein permeability and inflammation in *E. coli* endotoxin-induced ALI and pneumonia in C57BL/6 mice, which might be due to the increased level of KGF mediated by expression of the mRNA from MSC MVs in the injured alveolus (107, 121). Similar findings were obtained in a study using an *ex vivo* perfused human lung injured with bacterial pneumonia, which showed increased AFC and reduced lung protein permeability following MSC-EVs administration (117). The presence of hepatocyte growth factor (HGF) in MSC-EVs has been verified to stabilize pulmonary endothelial barrier function, as evidenced by reduced endothelium permeability and apoptosis rate, as well as increased cell proliferation in LPS-induced endothelial cell model (122). Alveolar epithelium forms a tighter monolayer than the endothelium. Epithelial barrier dysfunction in ARDS facilitates accumulation of protein-rich edema fluid and inflammatory cells in alveolar cavity. Previous study noted that allogeneic human MSCs restored epithelial protein permeability across human AT II cells injured by cytomix (a combination of IL-1β, TNF-α, and IFN γ). This beneficial effect on human AT II cells was mediated by angiopoietin-1 (Ang1) release from MSCs to prevent actin stress fiber formation and claudin 18 disorganization following an inflammatory insult in part through suppression of NF-κB activity (123). MSC-EVs may also play a protective role in ARDS through mitochondria transfer. In the ARDS microenvironment, MSC-EVs could restore alveolar-capillary barrier integrity at least partially *via* mitochondrial transfer to rescue mitochondrial dysfunction (124).

### 3.3 Immunomodulatory

At present, the most widely recognized pathogenesis of ARDS is excessive and uncontrolled inflammatory response in the lung. As a promising cell-based therapy candidates, MSCs and their derived EVs hold great potential for their ability to modulate/suppress innate and adaptive immune systems in the treatment to various inflammatory disease, including ARDS (125). MSCs harvested from different tissues and their EVs could increase survival rate and attenuate systemic and pulmonary inflammation in animals suffering from lung injury induced by various insults, whether administered systemically or intratracheally (13, 54, 121, 126–130). During the early phase of ARDS, neutrophils are recruited into the alveolar space in response to the chemokines released by macrophages and epithelial cells, activated excessively and undergo delayed apoptosis. Nevertheless, neutrophil apoptosis is essential for the resolution of ARDS. The study found that administration of MSC-CM attenuated LPS-induced ALI by inducing neutrophil apoptosis, which might be related to the inhibition of NF-κB signalling pathway (131). Neutrophil extracellular traps (NETs) are structures formed by dying neutrophils as a novel defence mechanism against bacterial infections. However, excessive NETs formation is associated with exacerbated immune responses and causes damage to lung tissue (132). Treatment with MSCs could reduce the oxidative stress and release of NETs, thereby alleviating lung injury and improving survival in LPS-induced ALI of mice (133). Cholinergic anti-inflammatory pathway (CAP) activation has been identified as a critical mechanism underlying the therapeutic effects of MSCs on ARDS, which is mediated by MSC-derived PGE2 (134). MSCs-derived EVs carrier multiple cargoes, in which miRNAs are extensively studied and considered to modulate gene expression and function of target cells under pathological conditions. In a rat model of traumatic ALI, miR-124-3p transferred by MSC-derived EVs could improve oxidative stress injury and inhibit inflammatory response through directly targeting and downregulating purinergic receptor P2X ligand-gated ion channel 7 (P2X7), which was overexpressed in traumatic ALI rats (128). Macrophages are key innate immune cells responsible for ARDS inflammatory response. Recent study revealed that the highly expressed miR-181a in MSC-EVs in the ARDS environment was crucial for the EVs immunomodulatory effect on human macrophages exposed to LPS as well as the mouse model of LPS-induced lung injury *via* PTEN-pSTAT5-SOCS1 axis (135). A study conducted by Wang et al. demonstrated that MSC-EVs could alleviate ALI *via* transfer of miR-27a-3p to alveolar macrophages and promoting M2 macrophage polarization (136). Apart from miRNA, mitochondrial components packed in MSC-derived EVs are increasingly attracting researchers interests and widely studied in recent years. Human MSCs could enhance phagocytic capacity and promote anti-inflammatory phenotype in human macrophages stimulated with LPS or ARDS bronchoalveolar lavage fluid (BALF) *via* EVs mitochondrial transfer (137). Furthermore, EVs from adipose-derived mesenchymal stem cells (AdMSCs) could restore metabolic homeostasis of macrophages and promote a functional shift of LPS-stimulated macrophages from M1 proinflammatory to M2 anti-inflammatory phenotype *via* transferring mitochondrial components (138). Cytokine IL-27 has been identified as a pro-inflammatory factor in the pathology of sepsis, which is primarily released by dendritic cells and macrophages (139). It was revealed that IL-27 in BALF and serum was elevated in ALI/ARDS patients and associated with disease severity. Blocking IL-27 with neutralizing antibodies could reduce pulmonary inflammation and improve survival rate in a mouse model of cecal ligation and puncture (CLP)-induced lung injury. From this, IL-27 is a critical cytokine in ALI/ARDS and inhibition of IL-27 may represent a promising therapy for ALI/ARDS patients (140). Recent study revealed that EVs from adipose-derived MSCs could alleviate sepsis-induced lung injury in mice by inhibiting the secretion of IL-27 in macrophages (141). Metabolic pathways of macrophages are closely related to their activation state and function. M1 macrophages rely on glycolysis for energy production whereas M2 macrophages rely on oxidative phosphorylation (OXPHOS). Metabolism shift between glycolysis and OXPHOS is involved in M1/M2 macrophage polarization (142). It was reported that EVs derived from BMSCs could attenuate LPS-induced ARDS by modulating alveolar macrophage polarization through inhibiting cellular glycolysis (143). However, it should be noted that not all MSCs exhibit immunosuppression properties. Bone marrow MSCs contain different functional subpopulations, in which IL-17+ MSCs possess antifungal effect but fail to execute MSC-based immunosuppression owing to the downregulation of TGF-β mediated by the NF-κB pathway (112).

### 3.4 Inhibition of cell death

It is well-known that extensive apoptosis of the endothelial and alveolar epithelial cells is involved in the pathogenesis of ARDS patients, leading to alveolo-capillary barrier dysfunction and disability of lung edema fluid clearance by alveolar epithelial cells (2, 144–147). MSCs could be activated to reduce apoptosis in UV-irradiated fibroblasts and lung cancer epithelial cells, which may be explained in part by the upregulation and secretion of stanniocalcin-1 (STC-1) (148). Exosomal miR-22-3p from human umbilical cord blood-derived MSCs could suppress inflammatory reaction and pulmonary cell apoptosis rate by reducing frizzled class receptor 6 (FDZ6) in LPS-induced ALI, which further benefited from the upregulated miR-22-3p (149). Ferroptosis is a novel and iron-dependent programmed cell death (PCD) modality (150). Excessive ferroptosis contributed to intestinal ischemia/reperfusion-induced ALI (151). A study revealed that specific delivery of miR-125b-5p by adipose derived stem cells (ADSCs) EVs could alleviate the inflammation induced pulmonary microvascular endothelial cells (PMVECs) ferroptosis in sepsis induced ALI *via* regulating Keap1/Nrf2/GPX4 expression, thereby improve the acute lung injury (152).

### 3.5 Modulating autophagy

Autophagy is a highly conserved lysosomal degradation pathway that primarily functions as an adaptive role to maintain homeostasis of organisms at both basal and stress-induced increase levels (153). Diverse stimuli including bacterial infection, LPS exposure, sepsis, and mechanical ventilation, etc. have been shown to promote autophagy in multiple lung cell types and animal models of lung injury (154). However, the exact role of autophagy in ALI still remains controversial. Autophagy may play a protective or detrimental role in ALI depending on the underlying causes of lung injury and the stages of disease progression (154). Changes in autophagic flux caused by autophagy dysfunction may underlie acute lung injury during sepsis (155). It was discovered that BMSC-derived EVs could relieve LPS-induced acute lung injury through inhibiting autophagy stress in alveolar macrophages *via* delivering miR-384-5p to combine with Beclin-1 directly (156).

## 4 Functional optimization of MSCs and their derived EVs

Priming MSCs with various agents or methods was found to further improve their biological function and therapeutic efficacy (157). A study noted that ischemic preconditioning of MSCs for 60 min could potentiate their treatment effect on endotoxin-induced ALI (158). Hypoxic preconditioning promoted migration and survival capacity of MSCs through upregulating LincRNA-p21 (159). Data from Tsoyi et al. suggested that preconditioning with carbon monoxide improved efficacy of MSCs during sepsis by production of specialized proresolving lipid mediators (160). Administrating staphylococcal enterotoxin B (SEB)-preconditioned murine MSCs intravenously could also improve survival rates and bacterial clearance in the peritoneal fluids, blood and organs in a mouse model of sepsis (161). Additionally, sublethal exposure to LPS enhanced MSCs survival, antibacterial activity, and immune regulatory properties in septic mice (162). Moreover, pretreatment with cytokines further enhanced the capacity of MSCs to facilitate injury resolution following ventilator-induced lung injury (VILI) and pulmonary epithelial wound healing *via* a KGF-dependent mechanism (163). In addition to therapeutic effects presented by MSCs per se, MSCs can also serve as a vehicle for gene therapy to attenuate ALI/ARDS (**Figure 2**). Overexpression of various bioactive factors in MSCs was confirmed as an effective way to optimize their treatment capacity. Overexpression of TGFβ-1 in murine MSCs could improve lung inflammation and attenuate lung injuries by modulating the imbalance of Th17/Treg in LPS-induced ARDS mice (164). The expression of angiotensin-converting enzyme 2 (ACE2) protein decreased significantly in LPS-induced ALI mice, while treatment with MSCs overexpressing ACE2 further improved lung endothelial function and mitigated lung inflammatory response compared with MSCs alone (165). The administration of MSCs overexpressing the Ang1 gene greatly reduced pulmonary vascular endothelial permeability and inflammatory reaction in the lung after LPS exposure, which might be mediated by the down-regulation of TNF-α gene expression (166). The study by Wang et al. have introduced a novel MSC-based delivery system. They removed the nuclei of MSCs using density gradient centrifugation and genetically engineered them with homing molecules (CCR2, CXCR4, and PSGL-1) to enhance their tissue-specific homing properties. The results showed that these modified MSCs improved the delivery of anti-inflammatory cytokine IL-10 to target tissue compared to normal MSCs and control EVs, thereby alleviating inflammation and pancreatitis (167).

As an important mediator involved in intracellular communication, EVs derived from miR-30b-3p-overexpressing MSCs could inhibit the apoptosis of type II alveolar epithelial cells (ACEs) and protect against ALI in mice after LPS delivery through transferring miR-30b-3p to the recipient ACEs to downregulate the expression of serum amyloid A3 (SAA3), which was a target gene of miR-30b-3p and significantly up-regulated in ALI (144). MSC-derived EVs exhibit therapeutic effects on ALI/ARDS not only by inherent properties but also as natural drug delivery nano-carriers due to their specific structure. The methods of loading the therapeutic cargo into the EVs include active and passive encapsulation (77). Moreover, several approaches are used for EVs surface modification to make them a valid target-specific drug delivery system, including genetic engineering, covalent and non-covalent modification, among which genetic engineering of EV producing cells using plasmid vectors is a widely used approach for producing surface modified EVs (168). Gupta and his colleagues simultaneously constructed two different decoy protein receptors for the pro-inflammatory TNF-α and IL-6 on the surface of EVs from HEK293T cells and MSCs using an optimized surface-display technology. The engineered EVs were observed to be able to ameliorate systemic inflammation in three different inflammation models through inhibiting IL-6 and TNF-α signalling pathways (169). The construction of this decoy EVs may provide a new direction for ALI/ARDS therapy. In the context of COVID-19, EV-based therapy optimization strategies include screening and incorporating a set of miRNAs that can specifically bind to the coronavirus genome into EVs to destroy the virus, and combining the therapeutic properties of MSC-EVs with loading anti-viral drugs (77, 170).

## 5 Therapeutic effects of mesenchymal stem cells and their derived EVs on ARDS patients

Due to the promising therapeutic effect of MSCs and their derived EVs on ALI/ARDS exhibited in animal models, increasing clinical trials have been designed to examine their safety and efficacy in ALI/ARDS patients (**Tables 1**, **2**). A small sample size, randomized, placebo-controlled pilot study published in 2014 explored the safety and efficacy of allogeneic adipose-derived MSCs on ARDS. The results showed that systemic administration of allogeneic adipose-derived MSCs appeared to be safe and feasible in the treatment of ARDS. Levels of serum surfactant protein D (SP-D), an acute lung injury biomarker, were significantly lower at day 5 compared to day 0. However, the dosage of MSCs used in this clinical trial might be required to be optimized to improve efficacy (171). The STem cells for ARDS Treatment (START) trial, a phase 1 clinical trial conducted by Matthay et al., demonstrated that a single intravenous MSCs infusion was well tolerated in patients with moderate to severe ARDS, and no MSC-related adverse events were reported (172). In a randomized phase 2a safety trial as part of the START study, 40 patients with moderate to severe ARDS received either MSCs or placebo. The trial found no MSC-related predefined hemodynamic or respiratory adverse events, and there was no significantly difference in mortality between the MSC group and the placebo group (173). The larger scale clinical trials will be necessary to further assess the safety and efficacy of MSCs for ARDS treatment. However, the clinical application of MSCs is limited by their safety concerns, cell survivability, scalability and regulatory issues (174). MSC-derived EVs, as a novel, multitargeted, next generation biological agent and key paracrine factor, along with their superior safety profile, stability, and scalability, have emerged as a practical treatment option (174). Clinical trials aimed at exploring the safety and efficacy of MSC-EVs in the treatment of ARDS are currently underway (NCT04602104, NCT05354141). Patients infected with SARS-CoV-2 may develop ARDS in severe cases. Accumulating evidence demonstrated that patients with severe COVID-19 suffered from the hyperinflammatory syndrome or cytokine storm syndrome, characterized by hypercytokinaemia. Premised upon the antimicrobial and robust immunosuppressive property of MSCs and their derived EVs, they might inhibit SARS-CoV-2 theoretically and the resulting host inflammatory reaction (170). Recent studies have found that MSCs and their EVs are also effective in the treatment of COVID-19. An observational pilot study reported that the intravenous transplantation of MSCs improved the outcomes of patients with severe COVID-19 pneumonia (175). A small sample size randomized controlled trial aiming to determine safety and explore efficacy of MSCs infusion in subjects with COVID-19 ARDS have also demonstrated beneficial effect (176). A phase II randomized, multicenter clinical trial conducted by Zarrabi et al. enrolled 43 patients with COVID-19 induced ARDS, who were allocated into two intervention groups (MSC alone group, and MSC plus EV group) and a control group. Patients in MSC alone group received two consecutive doses of allogenic MSCs intravenously at a dose of 100×10^6^ ± 10%, while those in MSC plus EV group received one dose of MSCs (100×10^6^ ± 10% cells) intravenously followed by one dose of MSC-EVs (isolated from the 200×10^6^ ± 10% cells) through respiratory inhalation. There was an interval of 48 h between the first dose and the second dose in both groups. The results indicated that the systemic administration of MSCs and nebulized inhalation of MSC-EVs were safe and could significantly reduce the serum levels of inflammatory markers in COVID-19 patients with ARDS (177). However, results from a recently published randomized controlled trial including 222 patients with moderate to severe SARS-CoV-2-related ARDS indicated that intravenous infusion of MSCs, while safe, did not reduce 30-day mortality or improve 60-day ventilator-free days compared with placebo (178). In a prospective non-randomized open-label cohort study published in 2020, EVs derived from allogeneic bone marrow mesenchymal stem cells (ExoFlo™) were a safe, efficient, and promising therapeutic candidate for severe COVID-19 cases (174). This result was further supported by a randomized placebo-controlled dosing clinical trial. The study enrolled and randomized 102 patients with COVID-19 associated moderate-to-severe ARDS receiving 10 mL ExoFlo™, 15 mL ExoFlo™, or placebo on day 1 and 4. Although there was no statistically significant difference in all-cause 60-day mortality in the Intention-to-Treat (ITT) population, two doses of 15 mL ExoFlo™ safely reduced 60-day mortality rate as compared to the placebo in participants aged 18-65 with respiratory failure or moderate to severe ARDS using *post-hoc* subgroup analyses (179). Several randomized controlled trials designed to evaluate the safety and effectiveness of EVs in moderate to severe ARDS related to COVID-19 are now in progress (NCT05387278, NCT04798716, NCT04747574).

**Table 1:** Clinical Trials of the therapeutic effects of mesenchymal stem cells on ARDS patients.

Trial identification	Official title	Source of MSCs	Disease	Drug delivery protocol	Actual or estimated enrollment	Primary outcome	Phase	Status
NCT02097641	Treatment with allogeneic mesenchymal stromal cells for moderate to severe acute respiratory distress syndrome (START study): a randomised phase 2a safety trial	Human bone marrow	ARDS	10×10^6^ MSC/kg PBW, single dose, intravenously	60	The safety of the MSC infusion	Phase 2a	completed
NCT01775774	Mesenchymal stem (stromal) cells for treatment of ARDS: a phase 1 clinical trial	Human bone marrow	ARDS	Dose-escalation (1 million cells/kg PBW, 5 million cells/kg PBW, 10 million cells/kg PBW), single dose, intravenously	9	The incidence of prespecified infusion-associated events and serious adverse events	Phase 1	Completed
NCT01902082	Treatment of acute respiratory distress syndrome with allogeneic adipose-derived mesenchymal stem cells: a randomized, placebo-controlled pilot study	Human adipose	ARDS	1×10^6^ cells/kg body weight, single dose, intravenously	12	The occurrence of adverse events	Phase 1	Completed
NCT04713878	An 8-Week Trial of Mesenchymal Stem Cells Therapy in Patients With COVID-19 Pneumonia	/	COVID-19 Pneumonia	Day 0: 1 million cell/kg Day 2: 1 million cell/kg Day 4: 1 million cell/kg Intravenously	21	Improvement of clinical symptoms Reduction of cytokine storm	Not Applicable	Completed
NCT04355728	Umbilical Cord-derived Mesenchymal Stem Cells for COVID-19 Patients With Acute Respiratory Distress Syndrome (ARDS)	Human umbilical cord	COVID-19 ALI/ARDS	The first infusion: administered at 100×10^6^ cells/infusion intravenously within 24 hours of study enrollment The second infusion: administered within 72 hours of study enrollment	24	Pre-Specified Infusion Associated Adverse Events	Phase 1 Phase 2	Complete
NCT04522986	An Exploratory Study of ADR-001 in Patients With Severe Pneumonia Caused by SARS-CoV-2 Infection	Adipose	COVID-19 pneumonia	1×10^8^ cells once a week, total four times intravenously	6	Adverse events	Phase 1	Completed
NCT04371393	A Randomized Trial of Mesenchymal Stromal Cells for Moderate to Severe Acute Respiratory Distress Syndrome from COVID-19	/	COVID-19 ARDS	The first infusion: 2×10^6^ MSC/kg of body weight intravenously The second infusion: 4 days after the first infusion ( ± 1 d)	222	Reduction in all-cause mortality within 30 days after randomization	Phase 2/3	Complete
IRCT20200217046526N2	Allogenic mesenchymal stromal cells and their extracellular vesicles in COVID-19 induced ARDS: a randomized controlled trial	Perinatal tissue	COVID-19 ARDS	Intravenous infusion of two consecutive doses of allogenic MSCs at a dose of 100×10^6^ ± 10% over 10-12 min	43	Adverse events	Phase 2	Completed

MSCs, Mesenchymal Stem Cells; PBW, Predicted Bodyweight; ARDS,: Acute Respiratory Distress Syndrome.

**Table 2:** Clinical Trials of the therapeutic effects of mesenchymal stem cell-derived EVs on ARDS patients.

Trial identification	Official title	Source of Exo	Disease	Drug delivery protocol	Actual or estimated enrollment	Primary outcome	Phase	Status
NCT04602104	A Multiple, Randomized, Double-blinded, Controlled Clinical Study of Allogeneic Human Mesenchymal Stem Cell Exosomes (hMSC-Exos) Nebulized Inhalation in the Treatment of Acute Respiratory Distress Syndrome	Allogeneic human mesenchymal stem cell	ARDS	Phase 1: hMSC-Exos low dose Phase 1: hMSC-Exos medium dose Phase 1: hMSC-Exos high dose Phase 2: hMSC-Exos dosage 1 Phase 2: hMSC-Exos dosage 2	169	Incidence of adverse reaction TTCI 28-day mortality	Phase 1 Phase 2	Recruiting
NCT05354141	Bone Marrow Mesenchymal Stem Cell Derived Extracellular Vesicles for Hospitalized Patients With Moderate-to-Severe ARDS: A Phase III Clinical Trial	Bone marrow mesenchymal stem cell	ARDS	Aerosol inhalation in 7 consecutive days Intravenous infusion	320	60-day All-cause Mortality	Phase 3	Recruiting
NCT05387278	Safety and Effectiveness of Placental Derived Exosomes and Umbilical Cord Mesenchymal Stem Cells in Moderate to Severe Acute Respiratory Distress Syndrome (ARDS) Associated With the Novel Corona Virus Infection (COVID-19)	Placental	COVID-19 ARDS	EV-Pure™ and WJ-Pure™ Intravenous infusion	20	The safety and efficacy of EV-Pure™ and WJ-Pure™ administration	Phase 1	Recruiting
NCT04798716	Mesenchymal Stem Cell Exosomes for the Treatment of COVID-19 Positive Patients With Acute Respiratory Distress Syndrome and/or Novel Coronavirus Pneumonia	Mesenchymal stem cells	NCP	Intravenously every other day on an escalating dose: (2:4:8) Intravenously every other day on an escalating dose (8:4:8) Intravenously every other day (8:8:8)	55	The number of participants with treatment-related-adverse events The number of IMV days for patients	Phase 1 Phase 2	Not yet recruiting
NCT04493242	Bone Marrow Mesenchymal Stem Cell Derived Extracellular Vesicles Infusion Treatment for COVID-19 Associated Acute Respiratory Distress Syndrome (ARDS): A Phase II Clinical Trial	Bone marrow mesenchymal stem cells	COVID-19 ARDS	Experimental dose 1: Intravenous administration of normal saline 90 mL and ExoFlo 10 mL Experimental dose 2: intravenous administration of normal saline 85 mL and ExoFlo 15 mL	102	60-day mortality rate	Phase2	Completed
NCT04747574	A Phase I Feasibility Study to Evaluate the Safety of CD24-Exosomes in Patients With Moderate/Severe COVID-19 Infection	T-REx™-293 cells engineered to express CD24 at high levels	COVID-19	Aerosolized in normal saline for inhalation *via* a standard hospital-grade inhalation device, QD for 5 days, four dose escalation	35	Adverse events	Phase 1	Unknown
NCT04276987	A Pilot Clinical Study on Aerosol Inhalation of the Exosomes Derived From Allogenic Adipose Mesenchymal Stem Cells in the Treatment of Severe Patients With Novel Coronavirus Pneumonia	Adipose mesenchymal stem cells	NCP	5 times aerosol inhalation of MSCs-derived EVs (2.0*10E8 nano vesicles/3 ml at Day 1, Day 2, Day 3, Day 4, Day 5)	24	AE and SAE TTIC	Phase 1	Completed
NCT04491240	The protocol of Evaluation of Safety and Efficiency of Method of Exosome Inhalation in SARS-CoV-2 Associated Two-Sided Pneumonia	/	NCP	EXO 1 inhalation: Twice a day during 10 days inhalation of 3ml special solution contained 0.5-2×10^∧^10 of nanoparticles (EVs) of the first type EXO 2 inhalation: Twice a day during 10 days inhalation of 3ml special solution contained 0.5-2×10^∧^10 of nanoparticles (EVs) of the second type	30	Non-serious and Serious Adverse Events During Trial Non-serious and Serious Adverse Events During Inhalation Procedure	Phase 1 Phase 2	Completed
NCT04602442	The Extended Protocol of Evaluation of Safety and Efficiency of Method of Exosome Inhalation in COVID-19 Associated Two-Sided Pneumonia	/	NCP	EXO 1 inhalation: Twice a day during 10 days inhalation of 3ml special solution contained 0.5-2×10^∧^10 of nanoparticles (EVs) of the first type EXO 2 inhalation: Twice a day during 10 days inhalation of 3ml special solution contained 0.5-2×10^∧^10 of nanoparticles (EVs) of the second type	90	Non-serious and Serious Adverse Events During Trial Non-serious and Serious Adverse Events During Inhalation Procedure	Phase 2	Unknown
NCT04493242	Bone Marrow Mesenchymal Stem Cell Derived Extracellular Vesicle Infusion for the Treatment of Respiratory Failure from COVID-19: A Randomized Placebo Controlled Dosing Clinical Trial	Bone marrow mesenchymal stem cell	COVID-19 ARDS	ExoFlo-10: infusion of 10 mL ExoFlo mixed with 90 mL NS intravenously over 60 minutes on Day 1 and 4 ExoFlo-15: infusion of 15 mL ExoFlo mixed with 85 mL NS intravenously over 60 minutes on Day 1 and 4	102	All-cause 60-day mortality rate	Phase 2	Completed
IRCT20200217046526N2	Allogenic mesenchymal stromal cells and their extracellular vesicles in COVID-19 induced ARDS: a randomized controlled trial	Mesenchymal stromal cells derived from perinatal tissue	COVID-19 ARDS	First dose: intravenous infusion of allogenic MSCs at a dose of 100×10^6^ ± 10% Second dose: nebulized inhalation of MSC-EVs (isolated from the 200×10^6^ ± 10% cells) 48 h after the first injection	43	Adverse events	Phase 2	Completed

EVs, Extracellular Vesicles; TTCI, Time to Clinical Improvement; NCP, Novel Coronavirus Pneumonia; IMV, Intermittent Mandatory Ventilation; AE, Adverse Reaction; SAE, Severe Adverse Reaction; NS, Normal Saline.

Given that the results from published clinical trials have fallen short of expectations, it may be necessary to design larger scale, multicenter, and long-term follow-up clinical trials to assess the potential benefits of MSCs and their derived EVs for ARDS in the future. At present, two distinct subphenotypes of ARDS have been identified, termed hyperinflammatory and hypoinflammatory (180, 181). Different ARDS phenotypes and immune status of patients may affect the treatment response to MSCs and their derived EVs. Consequently, further studies are needed to determine whether there is a contrast in efficacy between patients with different ARDS phenotypes receiving MSCs and their derived EVs therapy. Reducing heterogeneity among enrolled patients with ARDS in clinical trials may also help improve the possible clinical benefits of MSCs and their derived EVs in the treatment of ARDS. Approaches to reduce heterogeneity in clinical trials include selecting patients for both phase 2 and 3 trials on the basis of physiological, radiographic, and biological criteria as well as exclusion of patients with significant comorbidities (182). With regard to COVID-19-induced ARDS, it has been demonstrated that aging may be associated with uncoordinated adaptive immune responses and poor disease outcomes (183). Therefore, higher doses or longer infusion of MSCs and their derived EVs may be required to achieve therapeutic benefit in the aged individuals. This aged-related dose-effect relationship should be further investigated in the future clinical trials. Additionally, the optimal administration route, dosage, frequency and time window for MSCs and MSC-EVs therapy are also issues that need to be addressed. Functionally optimized MSCs have not yet been applied in clinical trials, but in the future, it may be possible to attempt to apply functionally optimized MSCs and EVs to explore their therapeutic effects on ARDS.

## 6 Challenges and limitations

Despite numerous advantages, the clinical transformation and application of MSCs and their derived EVs remain to be facing many challenges and unsolved problems. One of the major hurdles is the technology of large-scale generation of MSCs. MSCs will gradually age and lose multipotency and proliferative capacities over continuous passages, which have a negative impact not only on the therapeutic effect themselves but also on the yield and quality of EVs. To overcome the limitation, one potential alternative is to derive MSCs from induced pluripotent stem cells (iPSCs) generated by somatic cell reprogramming. Compared with tissue-derived MSCs, iPSC-MSCs have shown superior proliferative capacity and immunomodulatory function even after 10 passages (184). Consequently, further investigations should focus on developing more efficient and cost-effective protocols for large-scale production of MSCs. Besides, heterogeneity of MSCs originated from different donors, cell isolation techniques, and cell culture environment also significantly influence the clinical application of MSCs, so it is imperative to strengthen the quality control of stem cell preparations (104). The yield, purity and quality control provision of EVs for clinical application remain a matter of much concern. At present, there are four main methods for isolating and purifying EVs: differential ultracentrifugation and density gradients centrifugation, size-exclusion-based method, precipitation, and immunoaffinity-based capture, among which differential ultracentrifugation is the most commonly used and reliable isolation method (185–187). This technique is simple and cost-effective, but also has many limitations, such as time-consuming procedure, non-vesicular macromolecule contamination and aggregation (186). Not only that, but huge differences in efficiency of EV recovery across research groups are reported (187). Nonspecific precipitation is an alternative method to sediment EVs using polyethylene glycol (PEG)-based solutions without the need for expensive ultracentrifuges. However, these sedimentation processes may not be appropriate for large scale production and may result in the co-isolation of nonexosomal vesicles (187). Using tangential-flow filtration to concentrate EVs based on their size is more promising than the sedimentation methods, yet the ultrafilters used are expensive and the co-isolation of material still exists (187). Immunoaffinity isolation, a method based on specific binding between antibodies and EV surface markers, seems to be the most promising method due to its superiority over ultracentrifugation in terms of product characterization and isolation (188). However, scaling up production when using this technology is still challenging. In summary, in the process of clinical translation, it is necessary to develop advanced and standardized platforms to improve EV purity and quantity. Combined sequential filtration with affinity-based chromatography may offer the best chance (187). Moreover, development of the optimal method for long-term preservation of EVs is also worthy of our attention (71). EVs are much more stable than MSCs at -80°C and can be stored for up to 1 year (77). Nevertheless, repeated freezing and thawing can lead to the EVs clustering and degradation of their cargo. Freeze drying formulations may be a good choice for preserving EVs at room temperature. A study noted that freeze-dried EVs performed better than the non-freeze-dried EVs both in terms of morphology and function (77).

## 7 Summary and future perspectives

The pathophysiology of ARDS involves multiple complex and overlapping mechanistic pathways, eventually triggering an exaggerated innate immune response in the lung, resulting in the diffuse alveolar damage characterized by neutrophilic alveolitis and hyaline membrane deposition (19, 189). As a multi-pathway targeted cell-based therapy, MSCs and their derived EVs show promise in the treatment of ALI/ARDS owing to their capacity of reducing lung vascular injury, restoring alveolar fluid clearance and switching the pro-inflammatory responses to a pro-resolving paradigm. However, despite promising preclinical and early clinical trial results, there is still a need for clinical studies with larger sample size and more rigorous design to clarify the role of MSC and their EVs in the treatment of ARDS. More importantly, current clinical trials have shown some variability in outcomes, which may be attributed to the heterogeneity of ARDS itself. Therefore, researchers should focus on and attempt to reduce the heterogeneity of ARDS in future clinical trials, which probably contributes to improve treatment outcomes.

ARDS is an inherently heterogeneous clinical syndrome, which can be categorized into subgroups based on clinical, physiological, radiographic imaging, and biological criteria (182). The clinical and biological heterogeneity of ARDS influences the responsiveness to treatment, which may explain why promising candidate therapies in preclinical experiments failed in human trials. Therefore, therapeutic discovery for ARDS requires new approaches that focus on the phenotypic and biological heterogeneity to target therapies toward specific subgroups of patients with ARDS (189). Precision medicine approach targeting patients on the basis of their molecular phenotypes of ARDS might help to develop more effective and individualized pharmacotherapies (190). Applying the platform trials, an innovative trial designs from other fields, to the study of ARDS can make more therapies tested in a time-efficient and cost-effective manner, and accelerate therapeutic discovery for ARDS (189). A deeper understanding of key nodes in mechanistic pathway involved in the pathogenesis of ARDS will facilitate the identification of therapeutic targets, ultimately reducing the mortality and morbidity in patients with ARDS.

## Author contributions

XZ wrote the first draft of the manuscript. LF, LL, and ZD drew the figures and tables. YJ and XY supervised the work and provided the comments and additional scientific information. JZ and FH reviewed and revised the text. All authors contributed to the article and approved the submitted version.
